# Imaging of an Inflammatory Injury in the Newborn Rat Brain with Photoacoustic Tomography

**DOI:** 10.1371/journal.pone.0083045

**Published:** 2013-12-26

**Authors:** Edgar Guevara, Romain Berti, Irène Londono, Ningshi Xie, Pierre Bellec, Frédéric Lesage, G. A. Lodygensky

**Affiliations:** 1 Department of Electrical Engineering, École Polytechnique de Montréal, Montréal, Québec, Canada; 2 Montreal Heart Institute, Montréal, Québec, Canada; 3 Research Center-CHU Ste-Justine, Department of Pediatrics, Université de Montréal, Montréal, Québec, Canada; 4 Geriatric Institute Research Center, Université de Montréal, Montréal, Québec, Canada; 5 Department of Computer Science and Operations Research (DIRO), Université de Montréal, Montréal, Québec, Canada; NIH, United States of America

## Abstract

**Background:**

The precise assessment of cerebral saturation changes during an inflammatory injury in the developing brain, such as seen in periventricular leukomalacia, is not well defined. This study investigated the impact of inflammation on locoregional cerebral oxygen saturation in a newborn rodent model using photoacoustic imaging.

**Methods:**

1 mg/kg of lipopolysaccharide(LPS) diluted in saline or saline alone was injected under ultrasound guidance directly in the corpus callosum of P3 rat pups. Coronal photoacoustic images were carried out 24 h after LPS exposure. Locoregional oxygen saturation (SO_2_) and resting state connectivity were assessed in the cortex and the corpus callosum. Microvasculature was then evaluated on cryosection slices by lectin histochemistry.

**Results:**

Significant reduction of SO_2_ was found in the corpus callosum; reduced SO_2_ was also found in the cortex ipsilateral to the injection site. Seed-based functional connectivity analysis showed that bilateral connectivity was not affected by LPS exposure. Changes in locoregional oxygen saturation were accompanied by a significant reduction in the average length of microvessels in the left cortex but no differences were observed in the corpus callosum.

**Conclusion:**

Inflammation in the developing brain induces marked reduction of locoregional oxygen saturation, predominantly in the white matter not explained by microvascular degeneration. The ability to examine regional saturation offers a new way to monitor injury and understand physiological disturbance non-invasively.

## Introduction

Periventricular leukomalacia (PVL) stands as a predominant cause of cerebral palsy and significant neurodevelopmental impairment in premature infants [1]. The primary physiopathological mechanism was thought to be secondary to hemodynamic instability in a pressure passive immature cerebral circulation weakening the watershed areas of the white matter composed of pre-oligodendrocytes highly vulnerable to free radicals [1]. Only recent epidemiological studies have shown an association between inflammation and white matter injury [2]–[4]. Yet it is not clear if inflammation induces hemodynamic changes in the developing white matter. An intravenous dose of LPS sufficient to cause hypotension also affected O_2_ delivery to white matter [5]. Yet an experiment conducted in fetal sheep showed that small intravenous doses of LPS not sufficient to produce hypoxemia and systemic hypotension still triggered significant white matter injury [6]. To date, little is known regarding local changes in oxygen delivery with LPS induced inflammation. In rodents, one of the most robust and reproducible post-natal models consists in an injection of LPS into the corpus callosum. It replicates every aspect of periventricular leukomalacia including astrogliosis, microglial reaction, pre-oligodendrocyte cell loss, necrosis, apoptosis, hypomyelination [7], [8], hippocampal atrophy [9] and behavioral hallmarks of PVL [10]. Recent characterization of the model by magnetic resonance imaging (MRI) has shown striking similarities with what is seen in preterm infants with ongoing injury such as a reduction of the apparent diffusion coefficient in the white matter, increased T2 relaxation time constant, measurable ventricular dilation and increased radial diffusivity [11].

The regional changes seen in white matter injury of the preterm infants have been described using near-infrared spectroscopy (NIRS). This well-established bedside technique is limited by the absence of depth resolved information where for instance the presence of sub-dural hematoma or scalp edema seen in newborns might lead to inaccurate results. Moreover the technique cannot differentiate the cortex from the white matter or saturation extracted from the venous circulation. Although partial depth information can be obtained by using tomographic reconstruction methods [12], [13], they remain limited. Photoacoustic imaging is a depth-sensitive, non-invasive technique that combines the intrinsic contrast capabilities of optical imaging with the advantage of high spatial resolution of ultrasound [14],[15]. By illuminating tissue using a short laser pulse, a transient thermoelastic expansion occurs, generating an ultrasonic pressure wave detected by an ultrasound transducer [16]. Using more than one wavelength and spectroscopic information on hemoglobin, it has the potential to determine the locoregional oxygen saturation. In mouse pups, recent data shows that just 24 hours of hypoxic ischemic injury was sufficient to induce a 40% reduction of cerebrovascular density [17]. In this study, we sought to evaluate changes in locoregional oxygen saturation 24 h after LPS exposure, at the peak of the inflammatory injury, and determine if any changes detected *in vivo* would correlate with modification of the microvascular skeleton of the cortex and the white matter. Several functional imaging studies in the developing brain have been successful in identifying a physiological response to external tasks [18]–[20]. Despite the success of this approach requiring a complex setup, attention has shifted to brain mapping in resting conditions [21], an approach more suitable in neonates, where task-based functional magnetic resonance imaging (fMRI) scanning is challenging. Resting-state functional imaging (rsMRI) simplifies the experimental design, making longitudinal studies feasible in infants [21]–[23].

Since the discovery of spatially remote areas connected by spontaneous low frequency in the temporal domain with coherent fluctuations in the BOLD signal (<0.1 Hz) [24], a number of resting-state functional connectivity studies have been performed in infants [22], [23], [25]–[28]; although infants presenting PVL were excluded from those studies [26], [27]. Recently, Smyser et al. have specifically characterized the dramatic effect of white matter injury in the preterm infant on resting state activity at term equivalent [29]. Thus, early detection of the functional connectivity changes induced by PVL could reveal itself as a non-invasive tool to monitor newborns at risk and eventually identify the ones requiring neuroprotective therapy. Still performing routine MRI in unstable extreme preterm infants is challenging. Having depth sensitive data on resting state connectivity using a non-invasive technique that could be performed at bedside would be immensely useful, which is why we explored the potential of photoacoustic imaging to detect functional connectivity.

The objective of this study was to determine the impact of inflammation on the corpus callosum and the cortex both known to be affected by inflammation. Moreover the possibility of assessing resting-state functional connectivity using PAT imaging in immature rat pups was examined.

## Materials and Methods

### Ethics statement

All surgical procedures performed according to the recommendations of the Canadian Council on Animal Care, were approved by the Animal Research Ethics Committee of the Montreal Heart Institute and by the Institutional Committee for Animal Care in Research of the CHU Sainte-Justine Research Center. All the procedures were performed under isoflurane anesthesia, and all efforts were made to minimize suffering.

### Neonatal inflammatory injury

Nineteen postnatal day 3 (P3) Sprague-Dawley rat pups from four litters were used in this study (9.8±0.3 g weight, Charles River, Wilmington, MA); the rat pups were given *ad libitum* access to food and water. P3 rat pups are equivalent to very premature human infants between 24 and 28 weeks gestation [30]. In this work, we followed the same animal procedure as in previous studies [11] which was outlined by the group of Cai [7], [8] and briefly described below.

**Figure 1:**
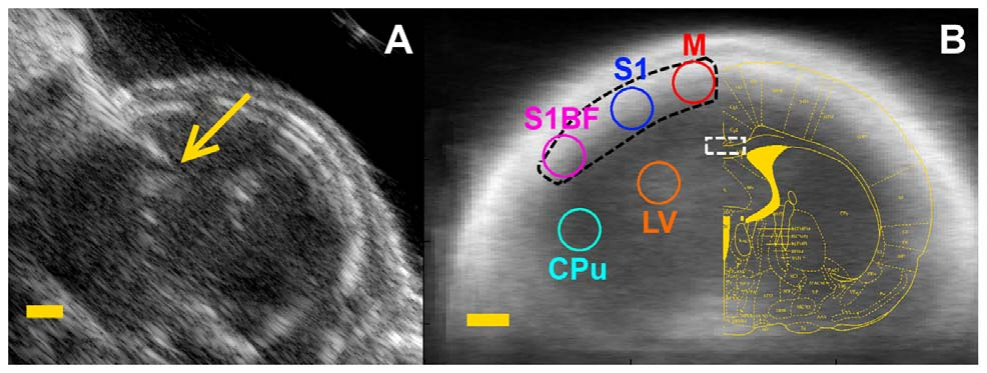
Coronal B-scan ultrasound images at the level of the LPS injection. (A) Arrow marks injection site. (B) Seeds placement, overlaid a coronal B-scan, averaged over 11 individual B-scans. Abbreviations: M, motor cortex; S1, primary somatosensory cortex; S1BF, barrel field primary somatosensory cortex; LV, Lateral ventricle; CPu, Caudate putamen. Black dotted line indicates Left Cortex ROI used for locoregional SO_2_ measurement. White dotted rectangle indicates ROI used for local SO_2_ measurement in corpus callosum. Scale bar: 1 mm.

P3 rat pups (n = 19) were randomized to two experimental groups: LPS (n = 11; n_male_  = 6, n_female_  = 5) and NaCl (sham n = 8; n_male_  = 7, n_female_  = 1). Pups were injected under ultrasound guidance using the Vevo LAZR micro-ultrasound system (FUJIFILM VisualSonics Inc., Toronto, ON, Canada) with LPS (1 mg/kg) suspension (E. Coli, serotype 055:B5, Sigma St Louis, MO) in 0.5 µL of sterile saline or with the same volume of sterile saline alone for the sham group. The injection was placed in the corpus callosum at the level equivalent to P-7, c9 [31], depicted in Figure 1A. Injections were made with a micropipette mounted on a microprocessor-controlled injector (Micro4 from World Precision Instruments) at a rate of 100 nL/min. All the injections were performed under isoflurane anesthesia, and all efforts were made to minimize suffering. All animals survived the injection. 24 hours later animals were imaged as described in the following sub-section.

### PAT imaging

The rat pups were secured in a custom-designed stereotaxic neonatal frame and placed on an imaging station; monitoring of the respiration and body temperature were done using the 1025L monitoring system (SA Instruments Inc., Stony Brook, NY). The Vevo LAZR photoacoustic imaging system was used to acquire all images with the LZ250 ultrasound probe at a central frequency of 21 MHz. The chosen coronal B-scan was located at the injection site. The acquisition time was 1.4 s per B-scan, with a field of view (FOV) of 13 mm width ×18 mm depth (512 A-lines, 416 samples) using a 256-element array transducer that provides 75 μm axial resolution. A time-series of B-scans were acquired in resting-state conditions for a 5-minute period. Although skin removal has been shown to improve image quality [32], it was not deemed necessary in this case as the use of pale-skinned strain led to reduced light absorption and the soft undermineralized skull of pups allowed better ultrasound transmission. Functional photoacoustic imaging was performed at two wavelengths (λ_1_ = 750 nm and λ_2_ = 850 nm) with pulse energy output of approximately 18 mJ/cm^2^. Total hemoglobin (HbT) and oxygen saturation (SO_2_) weighted images were internally computed from the raw data according to the procedure described in detail in refs. [33], [34]:
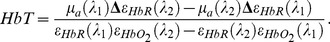
(1)

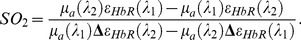
(2)where *μ_a_* was the estimated effective absorption coefficient (cm^−1^); 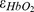
and 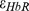 are the known molar extinction coefficients (cm^−1^M^−1^) of oxy- and deoxygenated hemoglobin respectively; and 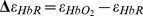. Once the effective absorption coefficients were estimated, HbT and SO_2_ could be calculated. Values of oxy- and deoxygenated hemoglobin (HbO_2_ and HbR respectively) were computed using the following equations:



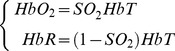
(3)One session of five minutes was recorded for every animal. The same recording session was used to assess both locoregional cortical saturation and functional connectivity. Locoregional cortical saturation was computed as the mean SO_2_ values during resting-state after removing frames contaminated with excessive motion (See Scrubbing technique below). Skin can be an issue in PAT imaging, especially when dark, hence the use of white Sprague-Dawley pups, which also have very thin not fully calcified skulls due to their age allowing high-quality data acquisition. SNR analysis was performed on the ROIs to make sure they consistently had at least 30:1 SNR. Average locoregional saturation values in corpus callosum were computed over a 1×0.5 mm^2^ rectangle; locoregional SO_2_ in the cortex was calculated over a closed spline that encircled the region of interest, either the left or right cortex, as shown on Figure 1B.

### Seed-based functional connectivity analysis

First, all functional images (PAT) within each scan were realigned to the first image to reduce the impact of motion artifacts. Images of each contrast were spatially smoothed with a Gaussian kernel of FWHM = 0.2 mm. Imaging of the anatomy was performed by an ultrasonic B-scan simultaneously recorded, from which the region corresponding to brain was manually selected using a closed spline curve to create a brain mask. All further processing was performed only on those pixels belonging to the brain mask. The image time series were temporally band-pass filtered (zero phase-shift fourth-order Butterworth filter) at 0.009–0.08 Hz, according to previous functional connectivity studies [35]–[38]. In order to account for spurious variability, several nuisance variables were regressed from each pixel time course including (a) The signal averaged over the lateral ventricles (LV), (b) the signal averaged over a region in the deep white matter and (c) three parameters obtained by rigid body motion correction (translation along X and Y axes and rotation around those same axes). Regression of the aforementioned parameters was performed simultaneously using a General Linear Model (GLM) from the package Statistical Parametrical Mapping [39] (SPM8, www.fil.ion.ucl.ac.uk/spm) running on a MATLAB (The MathWorks, Natick, MA) platform. In previous studies, the global brain signal, created from the average of all the pixels time traces, was found to introduce artificial anticorrelations [40], even though a neural basis for such anticorrelations is probable [41], it was not included as a regressor in this study.

All seeds were placed *a priori* on the rat brain using anatomical definitions from the Ramachandra atlas with complementary information from the Paxinos atlas [31], [42], as shown on Figure 1B. Seed time-courses were computed as the mean time course of the pixels within a 0.5 mm radius from the seed locus. Several seed radii were explored (from 0.1 to 0.5 mm), only limited by anatomical specificity; the largest radius was chosen because of stronger values in bilateral correlations.

### Scrubbing

Movement during scanning influences the quality of PAT data. Head motion causes spurious changes in photoacoustic signal intensity. After frame realignment and motion parameters regression, a technique called “scrubbing” was applied to remove measures distorted by unwanted movement [43]. Scrubbing relies on two measures to flag frames contaminated by excessive motion: framewise displacement (FD) and DVARS (D stands for temporal derivative and VARS standing for RMS variance over pixels) [23].

FD represents instantaneous head motion and is an empirical measurement computed from the motion parameters; in equation (4) 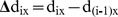 represents the derivative of translation along x and the same for the other rigid-motion parameter; the rotational displacement 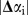
was converted from radians to mm by calculating arc length on a circle of radius 4.05 mm, which was approximately the mean distance from the cortex to the center of the head in P4 rats.
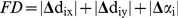
(4)


DVARS was computed as the RMS of the derivative of the global brain signal; this measure represents how much the intensity of a brain image changed in comparison to the previous frame.
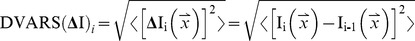
(5)


In equation (5) 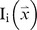 represents the image intensity at pixel *x* on frame *i*. A threshold is applied on FD and DVARS measures, and those frames over the threshold were flagged. After carefully reviewing the plots of the whole population, values of FD >0.001 mm and DVARS >3000 were chosen to represent levels above the norm in unmoving pups. A minimum of 80% of each session (∼4 minutes) of utilizable PAT data was further required for inclusion in the results below. Non-flagged frames were then concatenated to perform bilateral correlation analysis. No harmful effects have previously been found with the use of discontinuous functional data in previous seed-based resting-state studies [43], [44]. Figure S1 shows an example of flagging the frames of suspect quality.

With the unilateral LPS injection we investigated bilateral differences between brain regions so the metric used to evaluate functional connectivity was a regional bilateral functional correlation, defined as the correlation between each seed time-course and its contralateral homologue, yielding six values for each rat pup. The Pearson’s coefficient r-values were converted to Fisher Z measures using 
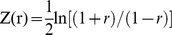
 before performing the population-level inferences.

### Histology

LPS and NaCl-injected brains were fixed after PAT imaging by transcardiac saline perfusion followed by 4% paraformaldehyde. The whole brains were kept at in paraformaldehyde 4°C and transferred to 30% sucrose after twenty-four hours. Cryosections (50 μm) were obtained and kept in cryoprotectant solution at −20°C.

### Lectin Staining and Vessel Counting

Four consecutive brain slices were chosen from each brain, spanning the injection site for staining and analysis with tetramethylrhodamine isothiocyanate (TRITC)-conjugated lectin to characterise microvasculature [45], [46]. The sections were pre-incubated at room temperature in 1% Triton X-100/PBS for 30 minutes, followed by a 2-hour incubation in the same solution with TRITC–conjugated lectin from Griffonia simplicifolia (1∶100; Sigma-Aldrich). Brain sections were subsequently subjected to three 10-minute washes with PBS and mounted on microscope slides. Sections were assessed using fluorescent microscopy (20x, Leica DMR with Retiga 1300 Qimaging Camera), and the microvascular skeleton was analyzed by AngioTool [47]. This approach was employed rather than a standard quantification of vessel surface. Indeed vessel thickness appearance was modified by the presence of macrophages also stained by lectin in the brains exposed to LPS. Fragmentation of microvasculature is not straightforward to determine solely by visual inspection, therefore robust imaging analysis tools are required. Twenty-eight images were taken for each brain covering three regions of interest (left cortex, right cortex and corpus callosum). Each image was then individually processed and analyzed using AngioTool to calculate the total and average skeleton lengths of microvessels in the regions of interests.

### Statistical analysis

Statistical significance in imaging and in microvasculature morphometry measures was evaluated using Wilcoxon-Mann-Whitney test. Since histology and locoregional SO_2_ measures were performed over only a few *a priori* planned regions, correcting for multiple comparisons was not done, according to recommendations from the literature [48], [49]. For seed based connectivity, a false discovery rate (FDR) multiple comparisons test was applied [50]. FDR-adjusted p-values were then considered significant at p<0.05. In order to find out if gender was a confounding factor, a Fisher’s exact mid-P method was used [51]. All data are presented as mean ± standard error of the mean (SEM).

## Results

### Locoregional oxygen saturation

**Figure 2:**
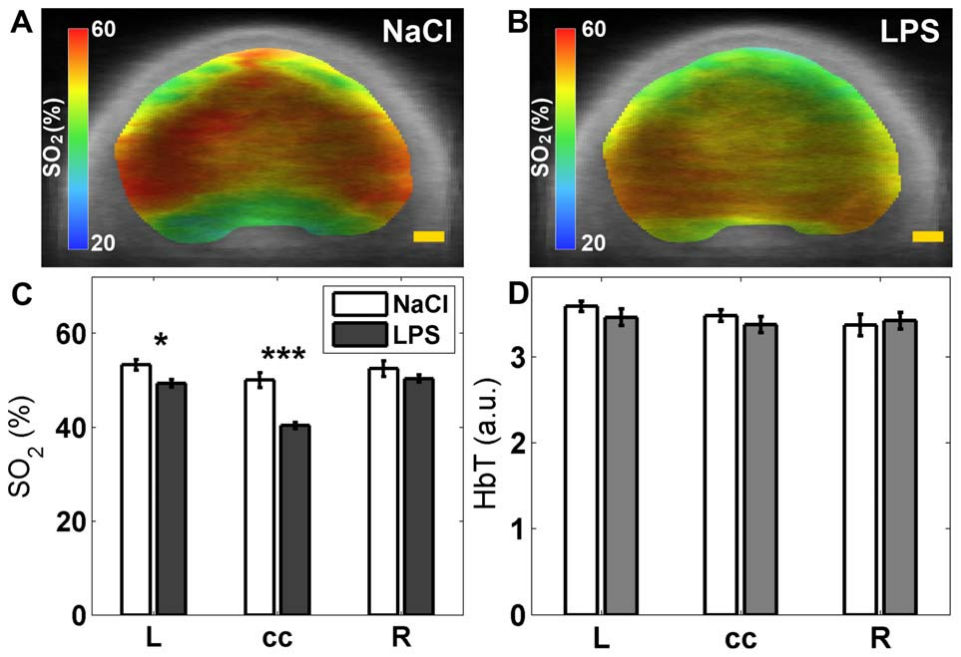
Locoregional cortical saturation (SO_2_%) following LPS exposure. (A) Group averaged SO_2_ weighted PAT image for sham controls (NaCl), (B) Group averaged SO_2_ weighted PAT image for LPS group, (C) SO_2_ Comparison performed between LPS group (N = 11), and NaCl (sham) group (N = 8). SO_2_ showed significant decrease in the LPS group compared to NaCl group in left cortex (L) and corpus callosum (cc). The hemisphere contralateral (R) to injection showed no difference. (D) HbT comparison between LPS and NaCl groups, no significant differences were found in any of the explored ROIs. Scale bar: 1 mm; *P<0.05, ***P<0.001.

After removal of four animals due to excessive motion (these animals were not included in the population description above) average SO_2_ was measured both on the left cortex (unilateral to LPS injection), the corpus callosum (cc) and on the right cortex. Group-averaged SO_2_ weighted images of sham controls and LPS are respectively depicted in Figure 2A and Figure 2B. Average SO_2_ values measured on the left cortex and the corpus callosum significantly decreased in LPS, when compared to sham controls, as shown on Figure 2C; more specifically, mean SO_2_ in the left cortex changed from 53.3% in the NaCl group to 49.4% in the LPS group (p = 0.0203). In the corpus callosum, mean SO_2_decreased from 50.1% in the NaCl group to 40.4% in the LPS group (p = 0.0002). Figure 2C shows no significant SO_2_ differences (p = 0.2060) on the right cortex (contralateral to the injection site). The association between gender and locoregional SO_2_ values was not statistically significant (p = 0.1089). No significant differences were found in locoregional HbT values, as depicted in Figure 2D.

### Microvascular quantification

**Figure 3:**
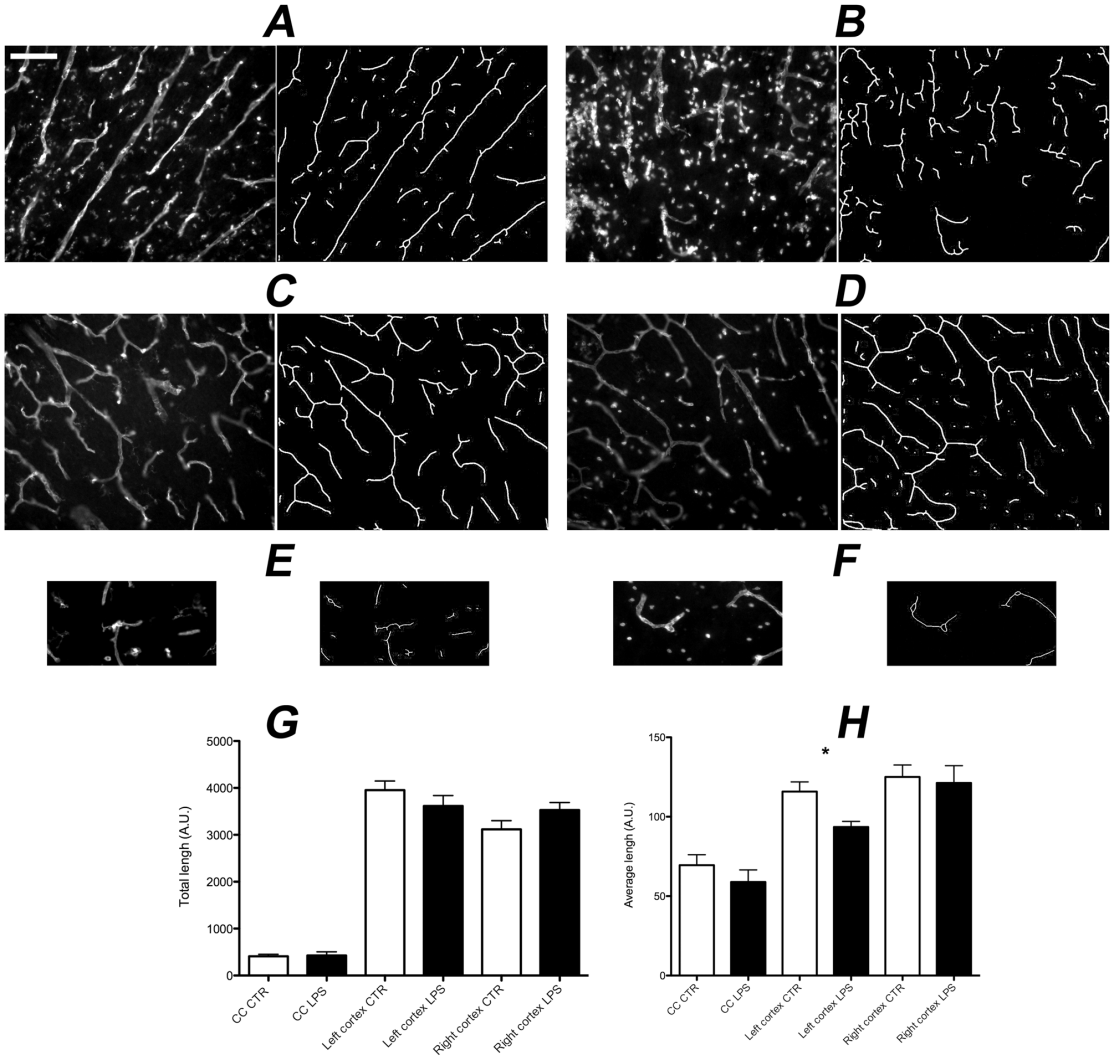
TRITC-conjugated lectin staining of brain sections 24h after LPS or saline injection with the associated microvascular skeleton extracted with Angio Tool. (A) left cortex; sham, (B) left cortex; LPS, (C) right cortex; sham, (D) right cortex; LPS, (E) corpus callosum; sham, (F) corpus callosum; LPS. The average microvessel length in the left cortex of brains injected with LPS (B) is reduced with vessels appearing fragmented compared to the brain injected with saline (A). Bar graphs of total microvessel length (G) and average microvessel length (H) in rat brain sections over the cortex and corpus callosum using the skeleton technique with AngioTool. Note the significant decrease in average microvessel length on the left cortex in brains injected with LPS (* p = 0.0093). Scale bar  = 100 μm.

We analyzed changes in vessel lengths in rat pup brains post LPS injection using lectin fluorescent staining due to its sensitivity in detecting microvessels [46]. We found that the average length of microvessels in the left cortex of the LPS injected brains were greatly reduced compared with the saline injected brains (*p* = 0.0093; Figure 3). We found no statistical difference in microvessel length in the right cortex and in the corpus callosum. Moreover, the average microvessels length in the left cortex was positively correlated with locoregional SO_2_ values measured with PAT imaging (r^2^ = 0.3011, *p = 0.0341).

### Seed-based functional connectivity

**Figure 4:**
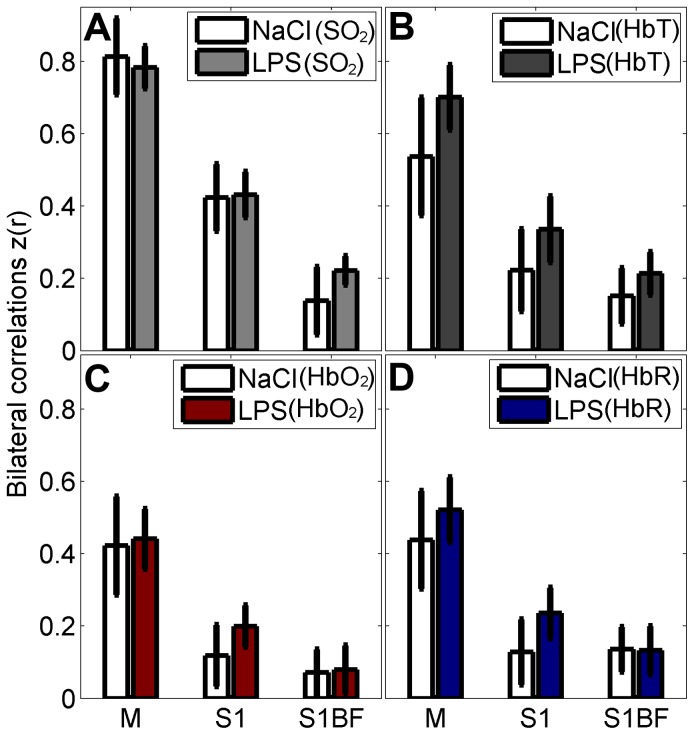
Regional bilateral functional correlation. Comparison performed between LPS group (N = 11), and NaCl (sham) group (N = 8); analysis done for every seed time-trace and its contralateral part. Contrasts shown: (A) SO_2_, (B) HbT (C) HbO_2_ and (D) HbR. Abbreviations: (M, motor cortex; S1, primary somatosensory cortex; S1BF, barrel field primary somatosensory cortex).

After scrubbing, a total of four scans were removed from the study due to excessive motion; (the same animals removed for computing locoregional SO_2_). The results displayed in Figure 4 show bilateral fc values getting smaller in seed pairs located farther from the top of the head. There was no statistical difference between both groups in all contrasts (SO_2_, HbT, HbO_2_ and HbR) though some displayed a consistent trend towards increased connectivity in the LPS group (HbT).

## Discussion

### Locoregional cortical saturation

Locoregional SO_2_ in the corpus callosum and on the cortex ipsilateral to the injection site were lower in the injured group, as compared to the sham group. This result coincides with lower regional cerebral tissue oxygen saturation found in preterm infants with transient PVL [52]. To understand if there was any anatomic substrate for these findings we studied the impact of LPS on microvasculature and found that only the left cortical average microvascular length at the site of injection was reduced. This reduction in average length can be explained by the fragmentation of the microvasculature, as a possible result of the endotoxin ability to damage vascular endothelium [53], [54]. When assessing the impact of inflammation on the cortex we found a significant correlation between non invasive PAT measures and histological assessments that explained 30% of the changes between cerebral microvascularisation rarefaction and reduction of saturation. This suggests that meso-scale measures of SO_2_, are only partly explained by visible microscopic changes. This reduction in regional saturation especially in the corpus callosum potentially contributes to the white matter injury seen in this model with, apoptosis, necrosis, hypomyelination and white matter loss [7]. The corpus callosum, which had the most significant reduction in saturation, had no measurable change in microvasculature integrity. A possible explanation may be a reflection of upstream effect of LPS on vascular resistance not evaluated by lectin staining, or subtle modifications of microvascular structure not recognized by immunohistochemistry. SO_2_ reduction may also be due to increased O_2_ consumption in this region [55]. This significant difference will be analyzed in future work using endoscopic two-photon microscopy to untangle the effect of LPS on capillary structure and regional blood flow. This underlines the importance of including measures *in vivo* of actual physiological changes not always identified by histology. The significant but smaller reduction at the left cortex may be explained by the fact that LPS does not have the same impact in sites farther from the injection locus and by selective vulnerability of white matter in the immature brain similarly to what has been extensively described in preterm infants [56]–[66].

Furthermore, these findings support the use of PAT as a non-invasive tool to study PVL animal models; due to its non-invasive and contrast agent-free nature, PAT could be applied to longitudinal studies either in adult humans [67] or in primates [68]. If applied to larger and more mature animals, limitations may arise and should be taken into consideration, such as the skin and skull thickness. Nevertheless human newborns [69] and some neonatal mammals such as dog puppies [70] have fontanels, potentially granting an accessible imaging window.

### Seed-based functional connectivity

When measuring bi-lateral seed connectivity, there was a strong correlation between the motor ROIs and less so in the S1 and S1BF ROIs and measured correlation values were similar that published in previous studies [38], [71]. Comparing groups, we observed no significant difference on resting state activity following LPS exposure. This might be explained by the young age of animals and the short interval between LPS exposure and PAT imaging. Previous studies have shown the presence of resting-state networks in preterm infants (from 29 weeks of gestation) [22], however the bilateral correlation values in motor and somatosensory cortices were weaker and more variable in the earlier weeks. The marked reduction of resting state activity in preterm infants white matter injury was described at term equivalent [29]. Little is known of acute modifications with injury in these infants. Our results thus show that while oxygen saturation is modified with injury between hemispheres, investigating the synchronicity of temporal fluctuations yielded no observable changes. One hypothesis is that the reduction of resting state activity in this population might lead to a large variability of measures across the population. A second hypothesis, which may combine with the first, is that the SNR of PAT imaging was not high enough to measure these small fluctuations homogenously in the brain. Indeed we found a reduction in bilateral correlations in the seeds located farther from the top of the skull attributed to lower SNR values in regions located further from the probe. We believe that resting state evaluation might be limited in these regions due to technical reasons. In future work, we will confirm these findings with rsMRI or invasive optical imaging based on intrinsic signals and laser speckle contrast imaging.

## Conclusions

The results of this exploratory work reveal lower locoregional SO_2_ values in LPS group, showing decreased values in the corpus callosum and in the left cortex, ipsilateral to the injection site. Our findings support the use of PAT as a completely non-invasive tool to assess average oxygenation values *in vivo* in the newborn brain.

## Supporting Information

Figure S1 **Framewise indices of data quality.** The dotted line in insets A and B represents the threshold to signal suspect frames: (A) Framewise displacement (FD) of head position and (B) DVARS measure. All frames surpassing the threshold in FD and DVARS time courses are flagged, generating a temporal mask: (C)Frames flagged as having a FD>0.001 mm and (D)Frames flagged as having a DVARS>3000. These temporal masks are augmented by also marking 1 frame back and 2 frames forward to accommodate temporal smoothing of PAT data: (E) Augmented FD mask and (F) Augmented DVARS mask. (G) Temporal mask comprised of the intersection of E and F.
(TIF)
