# Supplementary figures and images for: Imaging of an Inflammatory Injury in the Newborn Rat Brain with Photoacoustic Tomography
Source: PLoS One. 2013 Dec 26;8(12):e83045. doi: 10.1371/journal.pone.0083045 (PMC3873292; doi:10.1371/journal.pone.0083045)

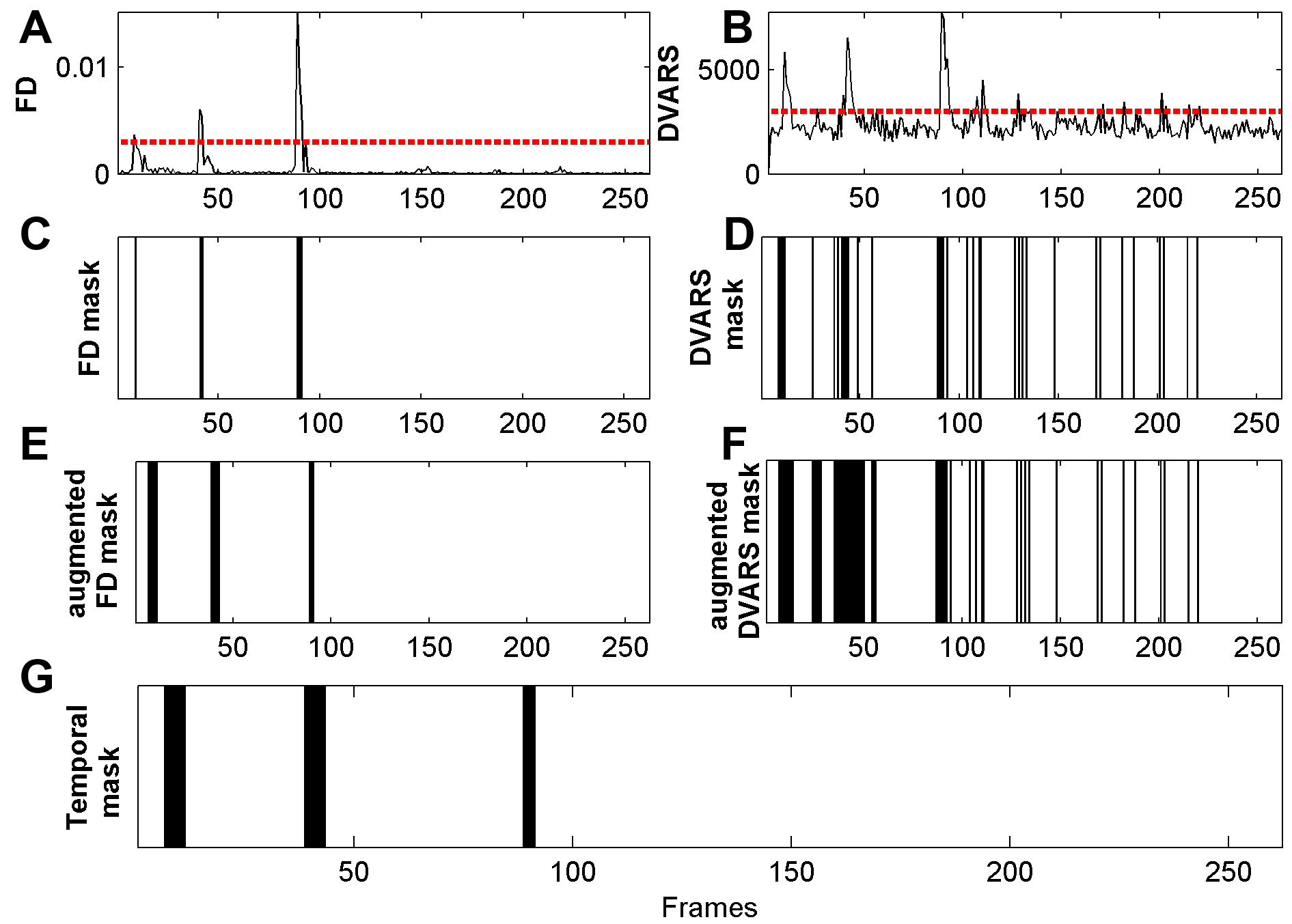

Supplement: Figure S1 — Framewise indices of data quality. The dotted line in insets A and B represents the threshold to signal suspect frames: (A) Framewise displacement (FD) of head position and (B) DVARS measure. All frames surpassing the threshold in FD and DVARS time courses are flagged, generating a temporal mask: (C)Frames flagged as having a FD>0.001 mm and (D)Frames flagged as having a DVARS>3000. These temporal masks are augmented by also marking 1 frame back and 2 frames forward to accommodate temporal smoothing of PAT data: (E) Augmented FD mask and (F) Augmented DVARS mask. (G) Temporal mask comprised of the intersection of E and F. (TIF) [file pone.0083045.s001.tif]
